# Skin Coloration Changes and Thermoregulation in *Anolis carolinensis* Across Different Thermal Environments

**DOI:** 10.3390/ani16020203

**Published:** 2026-01-09

**Authors:** Jiahui Hu, Yingying Xiong, Rui Liu, Xu Chen, Ai-Ping Liang

**Affiliations:** 1 Tianjin Key Laboratory of Conservation and Utilization of Animal Diversity, College of Life Sciences, Tianjin Normal University, Tianjin 300387, China; jhui11292023@163.com (J.H.); xyy202204666@163.com (Y.X.); liurui002927@163.com (R.L.); 2 Institute of Zoology, Chinese Academy of Sciences, Beijing 100101, China

**Keywords:** temperature, reflectivity, skin color change, thermoregulation

## Abstract

Ambient temperature plays a crucial role in shaping the skin color of some lizard species. While the long-term correlation between ambient temperature and skin color changes in lizards has been well-studied, how they adjust skin color and body temperature in response to short-term thermal fluctuations remains unclear. In this study, we examined the impacts of ambient temperature on the body temperature and skin color of *Anolis carolinensis*. In a white background, as the ambient temperature rose from 20 °C to 40 °C, both body surface and core temperatures increased; skin brightness rose from 71.47 to 88.05 cd/m^2^, chroma decreased from 43.55% to 36.43%, and hue dropped from 95.80° to 78.82°. Their changes against a brown background were similar to those against a white background. Correlation analysis showed that brightness was positively correlated with body temperature, chromaticity was negatively correlated with it, and hue negatively correlated with body temperature in white backgrounds but showed no significant correlation in brown backgrounds. As the ambient temperature rose from 20 °C to 40 °C, the spectral reflectance of skin in the visible (300–700 nm) and near-infrared (700–2500 nm) range increased from 26.01 ± 0.57% to 30.22 ± 0.63% and 8.61 ± 1.20% to 11.71 ± 1.48%, respectively. These results demonstrate that the skin color and spectral reflectance variations in *A. carolinensis* play a role in body temperature regulation. Additionally, this study offers new insights into the adaptive strategies of ectothermic organisms in balancing skin color and body temperature in fluctuating ambient temperatures.

## Simple Summary

Ambient temperature affects the skin color of some lizard species, but it was previously unclear how lizards adjust their skin color and body temperature in response to short-term temperature fluctuations. This study focused on the *Anolis carolinensis* to explore the impacts of different ambient temperatures on its skin color, body temperature, and skin spectral reflectance. The results showed that when the temperature rose from 20 °C to 40 °C, the lizard’s skin became brighter, color intensity decreased, and the reflectance of visible and near-infrared light increased. Around 30 °C was its optimal temperature, where body temperature was closest to ambient temperature. The study demonstrated that this lizard’s skin color changes not only serve camouflage and signal transmission but also help regulate body temperature. This provides a new perspective for understanding how ectothermic organisms adapt to ambient temperature fluctuations and has reference value for ecological conservation and biological adaptation research.

## 1. Introduction

Skin color change is a widespread adaptive phenomenon in nature, serving functions such as environmental matching (e.g., camouflage), thermoregulation, and intraspecific communication [1,2,3,4,5]. Chameleons, octopuses, and certain lizards (e.g., *Anolis* spp.) have emerged as ideal models for studying adaptive camouflage and color-changing mechanisms due to their ability to rapidly switch between multiple color patterns [6,7]. Meanwhile, as a key ecological factor, environmental temperature not only affects the metabolic rate [8] and biological rhythms of ectotherms but also exerts an influence on the body color of color-changing species [9]. Some studies suggested that lizards can optimize solar radiation absorption efficiency and maintain thermal balance through skin color regulation [6,10,11]. For instance, the ventral spot color of *Urosaurus ornatus* transitions from dark green to sky blue as temperature increases from 28 °C to 36 °C [10]. Similarly, the *Takydromus septentrionalis* exhibits a gradual lightening of skin color when deviating from its preferred temperature (30 °C), a process regulated by the G-protein signaling pathway and melanocyte-stimulating hormone (MSH) [11]. Population differentiation studies of the *Phrynocephalus putjatai* further demonstrate that the colorations of both dark and light lizards facilitated thermoregulation in their respective microhabitat simultaneously [5].

Previous studies have primarily focused on the effects of seasonal temperature changes on lizard body coloration. The modification of skin color changes its reflective properties, thereby contributing to the regulation of body temperature [12]. For instance, research on the high-altitude lizard *Sceloporus grammicus* has revealed that its dorsal skin reflectance (DSR, which is negatively correlated with body color darkness) decreases significantly during cold seasons [12]. Furthermore, DSR exhibits a negative correlation with heating rate—darker individuals achieve more efficient heat acquisition—and this finding supports the Thermal Melanism Hypothesis, reflecting the regulatory role of temperature-related environmental factors on the lizard’s body surface color over longer time scales [12,13].

Since over 50% of the energy in solar radiation is concentrated in the near-infrared reflectance (NIR) band [14], NIR reflectance may play a critical role in thermoregulation, specifically by reflecting NIR light to regulate temperature [15]. For example, arboreal anuran species (*Litoria caerulea*, *Agalychnis callidryas*, and *Hyla arborea*) reduce their thermal load through high NIR reflectance, enabling them to adapt to high temperatures in the forest canopy [15]. A rigorous electromagnetic model (the KKR method) was used to conduct optical simulations on the skin of reptiles like chameleons and iguanas, and the simulations revealed that nanostructures in the skin generate specific reflection peaks in the visible and UV regions, which in turn create the reptiles’ unique coloration [16]. However, there is still insufficient quantitative evidence regarding the role of color change in thermoregulation, and studies investigating the dynamic relationship between short-term temperature fluctuations and body color changes remain relatively limited.

**Figure 1:**
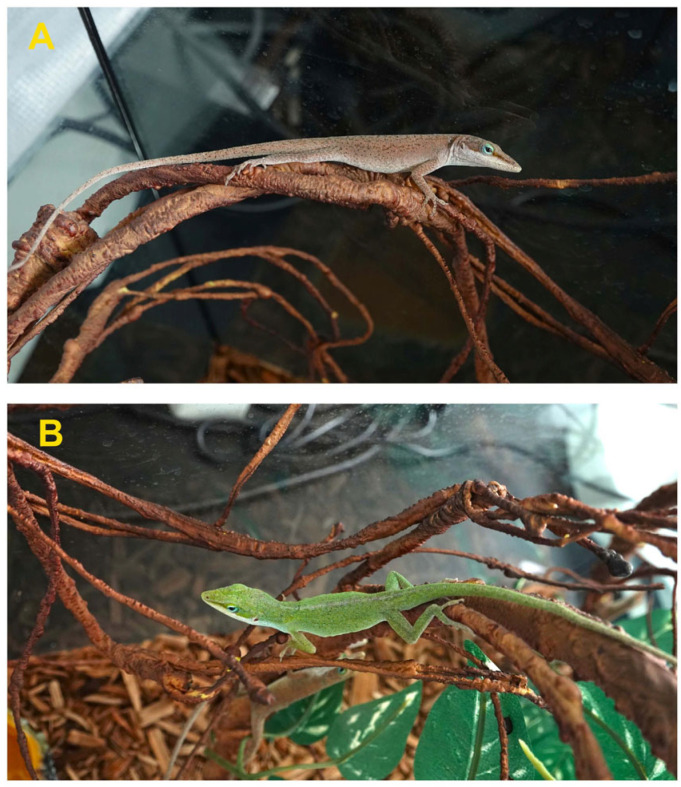
The skin colors of the *Anolis carolinensis*. (**A**) Brown; (**B**) Green.

The green anole (*Anolis carolinensis*) is a classic model species in ecological and evolutionary biology research [17]. This species has a critical thermal minimum (CTMin) of 11.25 ± 1.76 °C and a critical thermal maximum (CTMax, defined by the onset of convulsions) of 42.34 ± 1.53 °C [18]. *A. carolinensis* can rapidly switch its body color between bright green and dark brown (Figure 1). Its skin color may be affected by multiple factors. It exhibits different patterns in different habitats, and such rich color changes help it adapt to diverse habitats [19]. Multiple social behavior studies have shown that dominant males usually display brighter green, while subordinate individuals tend to turn brown [17].

Previous studies have shown that *A. carolinensis* does not hibernate but instead engages in facultative basking on sunny winter days, maintaining body temperatures that average 2.4 °C above ambient air temperatures while drastically reducing activity and movement to conserve energy [20]. Recent community-science data analyzed via computer vision further supports a seasonal association between color morph and temperature in wild populations. Brown coloration in *A. carolinensis* is significantly correlated with cooler temperatures [21]. This pattern suggests that color change may serve multiple, seasonally dependent functions, such as thermoregulation in winter and social signaling in summer. However, it remains unclear whether body color changes in response to ambient temperature over short periods.

In this study, we employed behavioral assays, image color analysis, and reflection spectroscopy to examine how body color and body temperature in *A. carolinensis* respond to changes in various ambient temperatures. Additionally, behavioral measurements were conducted in both white and brown backgrounds to assess whether shifts in body color and body temperature were driven primarily by temperature variation or by background adaptation. The research aimed to address three key objectives: (1) to determine whether skin coloration and body temperature change in response to ambient temperature variations; (2) to investigate the relationship between skin coloration and thermoregulation in this species; (3) to explore how shifts in skin spectral reflectance, including visible and near-infrared reflectance, contribute to thermoregulatory processes during the ambient temperature changes. We hypothesized that if darker coloration is a thermal adaptation to cooler conditions, it would correlate with lower temperatures on both backgrounds. By simultaneously measuring body temperature, we further examined whether color state correlates with thermal performance, thereby testing functional hypotheses of color change under controlled conditions.

## 2. Materials and Methods

### 2.1. Laboratory Rearing

The *A. carolinensis* (all experimental individuals were older than 9 months of age) used in this study were purchased from the commercial pet trade in Tianjin, China. Adult males exhibited a snout–vent length (SVL) of 59.69 ± 3.60 mm (*n* = 4), while adult females had an SVL of 51.67 ± 1.52 mm (*n* = 5). All individuals were housed in pet cages (50 cm × 30 cm × 35 cm). In the husbandry of experimental individuals, a metal mesh cover was affixed to the top of the enclosure to prevent escape. To ensure healthy bone development and overall wellbeing, full-spectrum fluorescent lighting with UV supplementation (UVB) (UVB 5.0 26W, REPTI ZOO, Dong Guan Etan Pet Supplies Co., Ltd., Dongguan, China) was installed within the enclosure to facilitate calcium absorption. The lamp was programmed on a 14 h light/10 h dark (14L:10D) photoperiod, with the light phase initiating at 07:00. A water basin was incorporated within each enclosure to uphold humidity levels. Daily misting was performed to sustain a relative humidity of approximately 50%, facilitating normal physiological functions. During the light phase, the temperature within the pet cages ranged from 27.10 ± 0.60 °C (in sheltered areas) to 29.05 ± 0.50 °C (directly under the UVB lamp). Each pet cage was furnished with appropriate bedding, shelters, and artificial branches for climbing. The lizards were provided and access to water and were fed crickets three times weekly, supplemented with a mixture of diced apples and vegetables.

### 2.2. Measurement of Skin Coloration and Body Temperature

#### 2.2.1. Skin Coloration Measurement

Under conditions ensuring regular physiological activity of the *A. carolinensis*, a series of temperatures (15 °C, 20 °C, 25 °C, 30 °C, 35 °C, 40 °C) was set using an artificial climate chamber (MGC-HP, Shanghai Yiheng, Shanghai, China) for testing. Thermocouples were used to measure the temperatures at the four corners and the intersection of the diagonals of the chamber. No significant temperature variation was observed inside the chamber, ensuring a stable ambient temperature. The tests were conducted in white (printing paper) and brown (pine bark) backgrounds. For background selection, we adopted the experimental protocol described by Smith et al. [22] and utilized a white background for color change assays. In contrast, a brown background was selected as it more closely mimics the chromatic characteristics of the natural habitats of *A. carolinensis*, ensuring ecological relevance for evaluating innate color-matching behavior. Prior to the test, a background radiation-corrected infrared camera (IRC; Model 248M, measurement precision ±2 °C or ±2%, FOTRIC, Shanghai, China) was employed to measure the time required for the lizard’s body temperature to reach thermal equilibrium under varying ambient temperatures. Based on our findings, the time it took for the lizard’s body surface temperature to achieve thermal equilibrium with ambient temperature ranged from 5 min to 25 min (Supplementary Materials Figure S1). In order to ensure the stability of the cloacal temperature (core body temperature) and body surface temperature, the environmental acclimatization time was uniformly extended to 60 min.

A digital single lens reflex (DSLR) (D610, Nikon, Tokyo, Japan) on a tripod with a gimbal, equipped with a prime lens (90 mm F/2.8 MACRO 1:1, Tamron, Saitama, Japan), was used for taking photos of lizards in manual mode (S:1/30, F:18, ISO 200). Lighting intensity, angle, and position within the chamber were consistent for each photograph to ensure uniform illumination [23]. In our experiments, the order of background exposure was fully randomized across individuals. Each lizard was acclimated to the test chamber under uniform conditions before being assigned to a background sequence; trials on different backgrounds were separated by a ≥24-hr rest period to minimize stress or residual effects. Before tests, each lizard was individually housed in a white rearing box (17 cm × 10.5 cm × 7.5 cm) and acclimated in the climate chamber for 1 h. During testing, dorsal photographs were taken every 10 min, and each lizard was photographed 5 times. For brown backgrounds, lizards were exposed to each temperature for 1 h, then the temperature was measured every 10 min, and body temperature was measured 5 times per individual. Three replicates were performed for each temperature in both backgrounds. In total, 810 photos were taken against a white background and 450 photos against a brown background (four individuals accidentally died during the experiment). Before each photography session, a 24-color checker (Aurora, X-Rite, Grand Rapids, MI, USA) was captured, and white balance calibration was performed in Adobe Lightroom Classic (Version 2024, Adobe, San Jose, CA, USA) using the software’s built-in auto-calibration function. Corrected images were saved in DNG (a universal raw file format). RGB values and luminance (Luminance = 0.299R + 0.587G + 0.114B) were extracted using the RGB Measure plugin in Image J (Version 1.54g, Java 1.8.0_345 (64-bit)) [7]. To quantitatively analyze head and dorsal coloration using the CIE LAB color space model, we computed calibrated red (R) and green (G) channels [(R − G)/(R + G + B)] and green-blue (G-B) channels [(G − B)/(R + G + B)] to establish a two-dimensional color representation. The Euclidean distance from the origin indicated chroma, while the angular position relative to the axes denoted hue, following methodologies outlined by Endler [24] and Smith et al. [22]. Physical chroma was determined as r = (x^2^ + y^2^) ^½, with hue angle calculated as θ = tan^−1^(y/x), where x and y represent standardized differences between R–G and G–B [22].

#### 2.2.2. Body Temperature Measurement

While the photos were being taken, the body temperatures of the lizards were recorded synchronously. A high-precision infrared thermometer (UT300S, measurement precision ±2 °C or ±2%, UNI-T, Dongguan, China) was used for measuring. For objects/animals ≥7 mm in width, the infrared thermometer provides biologically acceptable accuracy (difference ± within 1.0 °C) when measured at close range [25]. To verify the accuracy of the infrared thermometer, we compared body surface temperatures measured via the thermometer with those recorded by an infrared camera (Supplementary Materials Figure S2). The results showed that the infrared thermometer used in this study met the requirements of measurement accuracy.

The body temperature of *A. carolinensis* was measured at six different enclosure temperatures (15 °C, 20 °C, 25 °C, 30 °C, 35 °C, 40 °C), with temperature treatments consistent with Section 2.2.1. During testing, the surface body temperature was measured with the infrared thermometer every 10 min, and each lizard was measured 5 times. At the end of each test, the cloacal temperature (core body temperature) of the lizard was recorded using a thermocouple thermometer (DT-613, measurement precision ±0.5%±1 °C, CEM, Shenzhen, China), equipped with a calibrated thermocouple thermometer probe. The cloacal temperature measurement time was controlled within 5 s to avoid body temperature change. Three replicates were performed for each temperature in both backgrounds. In total, body surface temperature was measured 1260 times, and core body temperature 252 times. The lizards were given a 48 h recovery period between different temperature treatments.

Based on data characteristics and experimental representativeness (Supplementary Materials Figures S3 and S4), we finally selected three critical temperature nodes (20 °C, 30 °C, 40 °C) from six tests (15–40 °C) for in-depth analysis.

### 2.3. Measurement of Skin Spectral Reflectance

**Figure 2:**
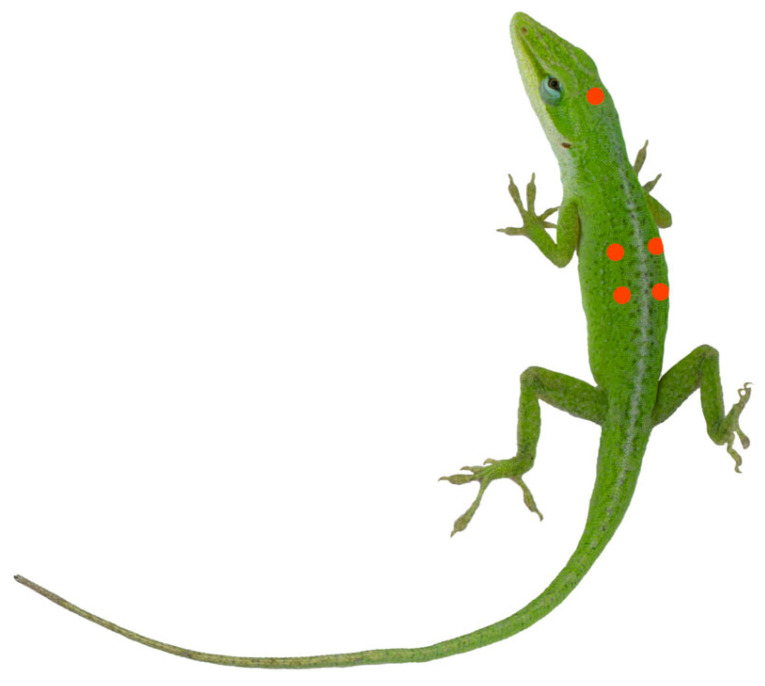
Schematic diagram of sampling points for skin spectral reflectance measurement in *Anolis carolinensis*. The red dots in the diagram are the locations selected for reflectance measurements.

Under conditions ensuring the normal physiological activity of *A. carolinensis*, a series of temperatures (15 °C, 20 °C, 25 °C, 30 °C, 35 °C, 40 °C) was set using an artificial climate chamber (MGC-HP, Shanghai Yiheng, Shanghai, China) for spectral reflectance testing, with all tests conducted against a white background. One site on the head and four sites on the dorsum were selected for the spectral reflectance measurements (Figure 2). After acclimating at each temperature for one hour, the skin spectral reflectance of *A. carolinensis* was measured in the UV-visible light range (300–700 nm) and the near-infrared region (700–2500 nm) using spectrometers (AvaSpec-ULS2048CL-EVO-UA-50; AvaSpec-NIR256-2.5-HSC-EVO, Avantes, Beijing, China), equipped with an optical fiber probe (FCR-19UVIR200-2-ME, Avantes, Beijing, China) and light source (AvaLight-HAL-S-Mini, Avantes, Beijing, China), calibrated with a spectral white diffuse reflectance standard (WS-2, Avantes, Beijing, China). The optical fiber probe was maintained 5 mm above the skin surface when measuring [22]. All devices were non-contact with the skin to minimize significant disturbance to the lizards, thereby preventing interference with experimental results. Nine lizards were used in the experiment, with each measurement point measured once per temperature group and three replicate experiments conducted for each lizard. In the end, a total of 405 spectral reflectance values were obtained.

To minimize interference from background noise, baseline drift, and stray light, denoising (median filtering) and smoothing (Savitzky–Golay filtering) were sequentially applied to the raw spectral data using the median and savgol_filter functions in Python 3.12 (SciPy library v1.11.4) [26,27]. A standardized dataset was thus generated for further analysis of the relationship between surface reflectance spectra, skin color characteristics, and temperature. Spectral preprocessing was performed using Python 3.12 (SciPy library v1.11.4) functions (midfield and savgol filter). Peak analysis of pre-processed spectral data was performed using Origin software (OriginPro 2021b SR1 v9.8.5.204, Origin lab, Northampton, MA, USA) to precisely identify wavelengths corresponding to peaks in the visible and near-infrared ranges (300–2500 nm). Subsequent statistical significance analysis of raw data at these characteristic wavelengths was performed to reveal differences among temperature groups. Additionally, Pearson correlation analysis was applied to the preprocessed spectral data to assess the relationship between near-infrared reflectance characteristics and temperature. In this study, *p* < 0.05 was considered to have statistical significance.

### 2.4. Data Processing

Temperature was used as the primary explanatory variable in this study, and data from 9 experimental individuals were integrated (each individual was measured in triplicate under each temperature condition). Data analysis was then performed to explore the relationships between temperature and coloration traits as well as body temperature indices. During the data analysis of skin color, body temperature, and reflectance at specific wavelengths, the Shapiro–Wilk test was applied to verify the normality of samples within each temperature interval: if the data met the normality assumption (*p* > 0.05), Repeated Measures ANOVA was used; otherwise, a nonparametric statistical method (specifically the Friedman test) was employed for intergroup difference analysis. In the paired analysis of surface temperature and core temperature, to ensure consistent data volume between the two temperature indicators, we first performed an averaging process on the repeated measurements of surface temperature for each experimental individual under each temperature condition. Normality of the processed data was then verified using the Shapiro–Wilk test: if the data satisfied the normality assumption (*p* > 0.05), a paired-samples *t*-test was used to evaluate the differences between surface temperature and core temperature under the two background conditions; if not, the nonparametric Wilcoxon signed-rank test was adopted for intergroup difference comparison.

The significance threshold for all statistical tests was set at α = 0.05. Data analysis and graph generation were performed using IBM SPSS Statistics (Version 27, International Business Machines Corporation, Chicago, IL, USA).

## 3. Results

### 3.1. Temperature-Dependent Changes in Skin Coloration and Body Temperature

#### 3.1.1. Variations in Skin Color Under Different Ambient Temperatures

**Figure 3:**
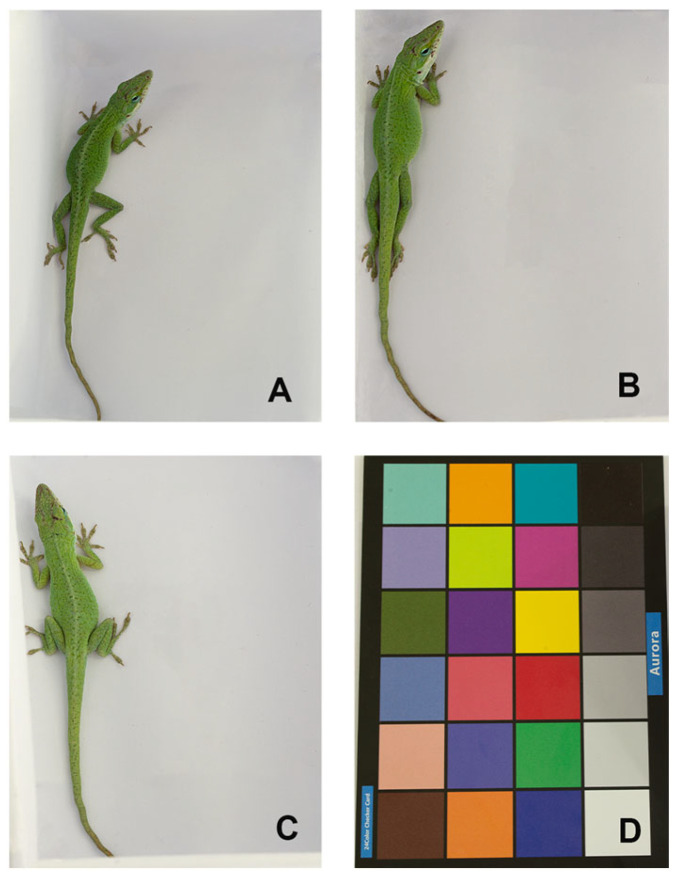
Skin color changes in the same *Anolis carolinensis* individual under different ambient temperatures. (**A**) 20 °C; (**B**) 30 °C; (**C**) 40 °C; (**D**) 24-color checkerboard.

**Figure 4:**
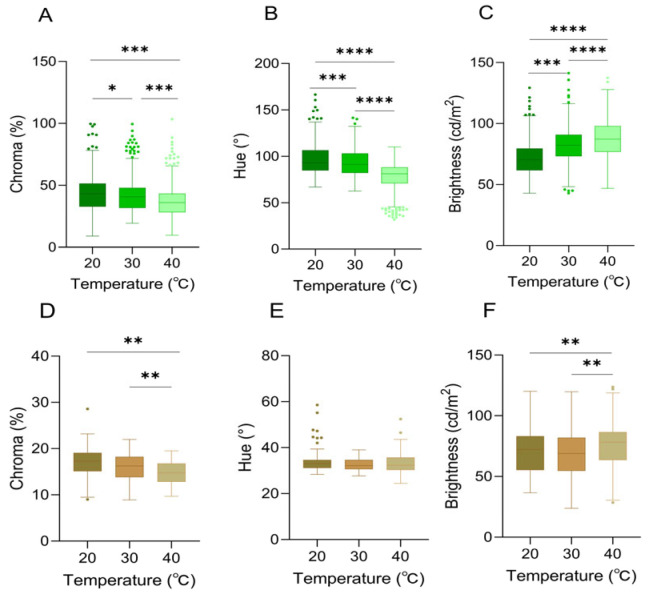
Skin color characteristics of *Anolis carolinensis* under different ambient temperatures. (**A**–**C**): White background; (**D**–**F**): brown background. (**A**,**D**) Chroma values; (**B**,**E**) hue values; (**C**,**F**) brightness values. There were significant differences between different boxes (Friedman test). Boxplots are represented by medians and whiskers (within 1.5 times of the interquartile range). The dots outside the box represent discrete values. Asterisks denote statistically significant differences (* *p* ≤ 0.05; ** *p* ≤ 0.01; *** *p* ≤ 0.001; **** *p* ≤ 0.0001).

When the background was white, the changes in chromatic space (brightness (B), chroma (C), and hue (H) values) of *A. carolinensis* skin color were significantly correlated with ambient temperature (Figure 3 and Figure 4).

There were significant differences in chroma among different ambient temperatures of 20 °C, 30 °C, and 40 °C (Friedman test, df = 2, *p* < 0.0001) (Table 1, Figure 4A).

Hue (H) was significantly associated with ambient temperature (df = 2, *p* < 0.001), and it exhibited a progressive decline in values across the thermal gradient. Notably, the maximum chromaticity was recorded at 20 °C (h° = 118.50° ± 3.20°), which corresponded to the most saturated green hue (Table 1, Figure 4B).

Within the ambient temperature range of 20–40 °C, a significant positive correlation between brightness (B) and temperature was identified (df = 2, *p* < 0.0001). Concurrently, the skin color exhibited a progressive transformation from dark green to light green (Table 1, Figure 4C).

The variation in color space (brightness (B), chroma (C), and hue (H)) of *A. carolinensis* was also significantly correlated with ambient temperature when the background was brown. Chroma (C) was significantly influenced by temperature (df = 2, *p* < 0.01), exhibiting a downward trend as temperature rose (Table 1, Figure 4D). A temperature-dependent distribution was demonstrated by hue (H), with a broader range being shown in the 40 °C group compared to the other groups (Table 1, Figure 4E). Brightness (B) peaked at 40 °C, indicating that skin color brightness was enhanced under high-temperature conditions (Table 1, Figure 4F).

#### 3.1.2. Variations in Body Temperature Under Different Ambient Temperatures

Under different environmental temperatures, the body temperature of this species does not exhibit sexual dimorphism (Supplementary Materials Figure S5). Therefore, data from both sexes were combined for subsequent data analysis.

**Figure 5:**
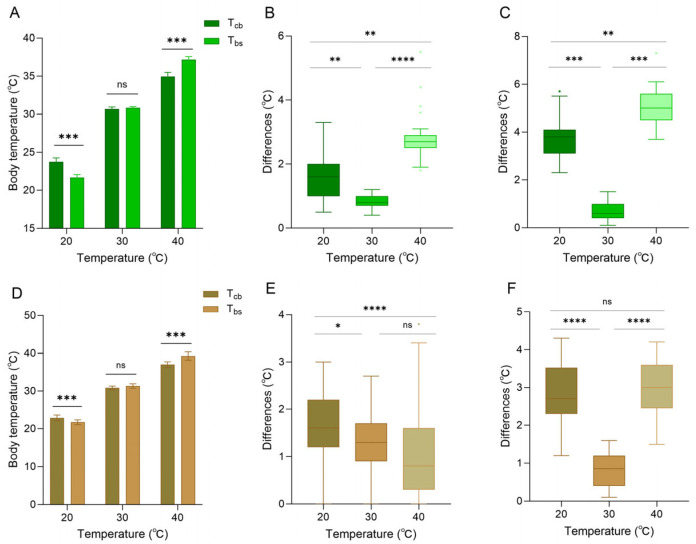
Body temperature of *Anolis carolinensis* at different ambient temperatures. (**A**–**C**) White background; (**D**–**F**) brown background. (**A**,**D**) Differences between core body temperature (T_cb_) and body surface temperature (T_bs_). (**B**,**E**) Differences between body surface temperature and ambient temperature. (**C**,**F**) Differences between core body temperature and ambient temperature. The difference in the histogram was statistically significant (paired samples *t*-test). The error line serves as an indicator of the standard error. There were significant differences between different boxes (Friedman test). Boxplots are represented by medians and whiskers (within 1.5 times of the interquartile range). The dots outside the box represent discrete values. Asterisks denote statistically significant differences (* *p* ≤ 0.05; ** *p* ≤ 0.01; *** *p* ≤ 0.001; **** *p* ≤ 0.0001) and ‘ns’, no significant difference (*p* > 0.05).

When the background was white, the skin color of the lizard was entirely green (based on RGB measurements). At 20 °C, core body temperature was higher than body surface temperature (paired sample *t*-test, t = 13.107, *p* < 0.001), and both were higher than ambient temperature (Figure 5A). At 30 °C, core body temperature was close to the body surface temperature (t = −1.633, *p* = 0.141), and both were close to the ambient temperature (Figure 5A). At 40 °C, body surface temperature was higher than the core body temperature (t = −22.913, *p* < 0.001), and both were lower than ambient temperature (Figure 5A). The differences between body surface temperature and ambient temperature at 20 °C, 30 °C, and 40 °C were 1.66 ± 0.73 °C, 0.83 ± 0.23 °C, and 2.84 ± 0.76 °C, respectively (Figure 5B). The differences between core body temperature and ambient temperature at 20 °C, 30 °C, and 40 °C were 3.74 ± 0.79 °C, 0.71 ± 0.39 °C, and 5.05 ± 0.80 °C, respectively (Figure 5C).

When the background was brown, the skin color of the lizard was entirely brown (based on RGB measurements). At 20 °C, core body temperature was significantly higher than body surface temperature (t = 9.489, *p* < 0.001), and both were higher than ambient temperature (Figure 5D). At 30 °C, body surface temperature began to exceed the core body temperature (t = −1.843, *p* < 0.076), and both were close to the ambient temperature (Figure 5D). At 40 °C, body surface temperature was higher than core body temperature (t = −6.043, *p* < 0.001), and both were lower than ambient temperature (Figure 5D). The differences between body surface temperature and ambient temperature at 20 °C, 30 °C, and 40 °C were 1.70 ± 0.64 °C, 1.31 ± 0.60 °C, and 1.02 ± 0.84 °C, respectively (Figure 5E). The differences between core body temperature and ambient temperature at 20 °C, 30 °C, and 40 °C were 2.85 ± 0.75 °C, 0.84 ± 0.45 °C, and 3.02 ± 0.73 °C, respectively (Figure 5F). It is worth noting that against a brown background, the difference between body surface temperature and ambient temperature was minimal at 40 °C. In contrast, the difference between core body temperature and ambient temperature at 40 °C was larger, but the difference was smaller than that of a white background at 40 °C (Figure 5).

### 3.2. Relationships Between Skin Coloration and Body Temperature

**Figure 6:**
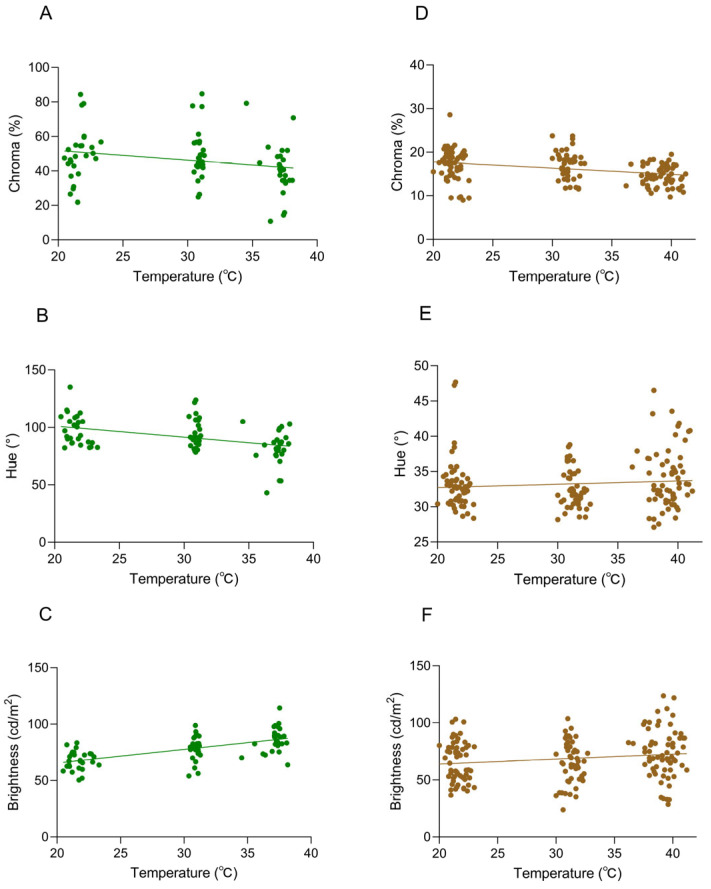
Correlations between body temperature and coloration in *Anolis carolinensis* at different ambient temperatures. (**A**–**C**) White background; (**D**–**F**) brown background. (**A**,**D**) Chroma values (Pearson’s r = −0.3022, *p* < 0.0001; Pearson’s r = −0.3976, *p* < 0.0001). (**B**,**E**) Hue values (Pearson’s r = −0.1505, *p* > 0.05; Pearson’s r = 0.04788, *p* > 0.05). (**C**,**F**) Brightness values (Pearson’s r = 0.6262, *p* < 0.0001; Pearson’s r = 0.1643, *p* = 0.0298). The dots in the graph represent the body temperature and chroma (hue/brightness) values of the individual at that ambient temperature, and the straight line is a linear regression line.

The body surface temperature showed significant differences with changes in environmental temperature, and multiple repeated measurements were conducted at each temperature treatment stage. Not only is the amount of data sufficient, but it is also highly consistent with the frequency and dimensions of the body color data collection. Therefore, in the analysis, we selected body surface temperature as the core response variable. When the background was white, a significant negative correlation was observed between hue and body temperature (Pearson’s r = −0.3022, *p* < 0.0001), suggesting that higher body temperatures corresponded to reduced chromatic expression (Figure 6A). Hue values decreased with rising body temperature, while chroma showed no significant association with thermal changes (Pearson’s r = −0.1505, *p* > 0.05; Figure 6B). A weak negative trend between chromaticity and body temperature was detected; however, the absence of statistical significance suggests no discernible association under the tested conditions. There was a significant positive correlation between brightness and body temperature (Pearson’s r = 0.6262, *p* < 0.0001) (Figure 6C). As the body temperature rose, the brightness value was significantly increased, and the correlation was highly statistically significant.

When the background was brown, a significant negative correlation between chromatic intensity and body temperature was observed (Pearson’s r = −0.3976, *p* < 0.0001) (Figure 6D), with chroma values decreasing significantly as body temperature increased. No significant correlation was observed between hue and body temperature (Pearson’s r = 0.04788, *p* > 0.05), indicating no meaningful association under the tested conditions (Figure 6E). There was a statistically significant positive correlation between brightness and body temperature (Pearson’s r = 0.1643, *p* = 0.0298) (Figure 6F). While the *p*-value (0.0298) fell below the conventional significance threshold (0.05), the correlation did not reach more stringent levels (*p* < 0.01), indicating a weak but detectable upward trend in brightness values with increasing body temperature.

### 3.3. Temperature-Dependent Variation in Visible and Near-Infrared Spectral Reflectance

**Figure 7:**
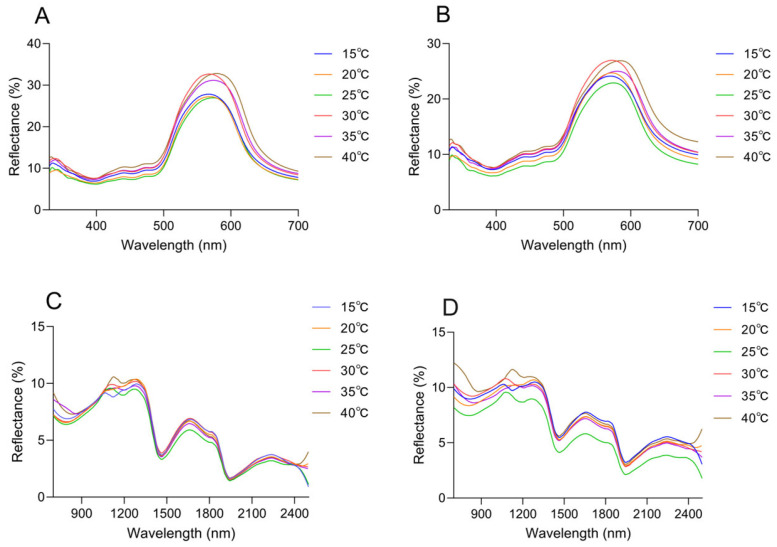
Spectral reflectance of the *Anolis carolinensis*’ skin under different ambient temperatures in a white background. (**A**) Dorsal reflectance at 300–700 nm. (**B**) Head reflectance at 300–700 nm. (**C**) Dorsal reflectance at 700–2500 nm. (**D**) Head reflectance at 700–2500 nm.

Changes in spectral reflectance of the dorsal and head surfaces were observed across the range of ambient temperatures. It peaked in the green spectrum range (580 nm), and subtle differences were presented in the curve shape (Figure 7A,B). The peak wavelength increased with rising temperature, reaching its maximum at 40 °C. The peak wavelength of the dorsal thorax rose from 567.17 nm at 15 °C to 578.87 nm at 40 °C, with reflectance increasing from 26.61 ± 0.66% to 31.40 ± 0.66%; the peak wavelength of the dorsal head increased from 569.51 nm to 584.71 nm, while reflectance rose from 23.62 ± 0.92% to 25.52 ± 1.41% (Table 2). Notably, under the same temperature conditions, a comparative analysis of the reflectance between the head and trunk reveals that the reflectance of the dorsal side of the head exhibits a significant difference (Supplementary Materials Figure S5).

**Figure 8:**
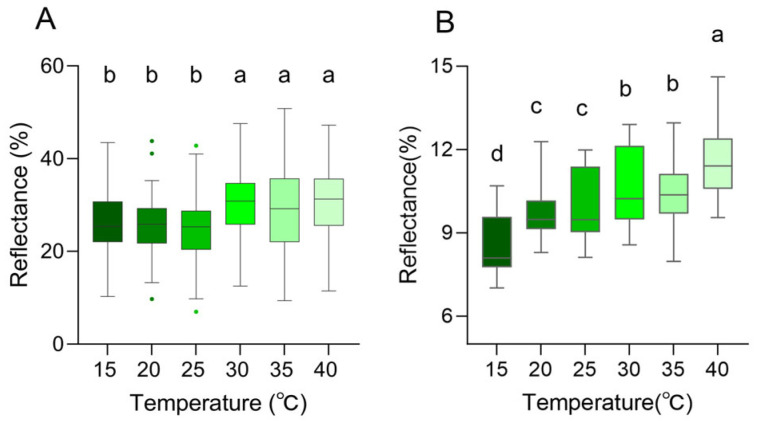
Skin spectral reflectance of the *Anolis carolinensis* at different ambient temperatures. (**A**) Peak reflectance in the 300–700 nm range. (**B**) Peak reflectance around 1100 nm. Boxes that do not share the same letter are significantly different from each other (*p* < 0.05) as determined by a Friedman test. Boxplots are represented by medians and whiskers (within 1.5 times of the interquartile range) the dots outside the box represent discrete values.

In the near-infrared range (700–2500 nm), the reflection curves of all skin regions were found to show a similar trend, and the reflection peaks were mainly observed at 1103.64 ± 31.69 nm, 1272.34 ± 11.65 nm, 1661.72 ± 3.71 nm, and 2228.28 ± 4.49 nm (Figure 7C,D). However, there were differences in peak reflectance between regions, with the head being higher than the dorsal overall. At 1103.64 ± 31.69 nm, the reflectance of the dorsal was 9.76 ± 0.50%, and that of the head was 10.46 ± 0.70%. At 1272.34 ± 11.65 nm, the reflectance was 10.02 ± 0.36% on the dorsal and 10.26 ± 0.69% on the head. At 1661.72 ± 3.71 nm, the reflectance was 6.60 ± 0.37% on the dorsal and 7.20 ± 0.71% on the head. At 2228.28 ± 4.49 nm, the reflectance was 3.53 ± 0.21% on the dorsal and 5.01 ± 0.59% on the head (Figure 7C,D, Table 2). There was no tendency for reflectance to increase as the temperature increased in 1272.34 ± 11.65 nm, 1661.72 ± 3.71 nm, and 2228.28 ± 4.49 nm (Figure 7C,D, Table 2). At 40 °C, both on the dorsal and on the head, there was always a high reflectance condition around 1100 nm; at 15 °C, the reflectance of the head and dorsal was always low at around 1100 nm (Figure 7C,D, Table 2). In the UV-visible range, the skin surface reflectance increased as the ambient temperature increased from 15 °C to 40 °C. Significant differences were observed between 25 °C and 30 °C. This was consistent with the colorimetric analysis results, which indicated higher reflectance corresponding to increased lightness (Figure 8A). Further statistical analysis of the NIR reflection results showed that there was a significant difference between 40 °C and other lower temperatures (15 °C, 20 °C, 25 °C) in the range of 1103.64 ± 31.69 nm (*p* < 0.05) (Figure 8B). There was also a significant difference between 15 °C and other temperatures (*p* < 0.05) (Figure 8B). Overall, it was observed that skin reflectance exhibited an upward trend as the ambient temperature increased (Figure 8B).

## 4. Discussion

Temperature-induced skin color changes exhibit unique regulatory mechanisms and functional priorities across different groups of ectothermic animals [10]. The evolution of color on the backs of color-changing lizards is driven by complex and variable climatic factors, and conducting targeted experimental tests is crucial to deeply analyze the various internal mechanisms that drive changes in animal coloration in different environments [28]. The present study reveals that changes in environmental temperature directly induce significant changes in body color: as temperature increases, brightness rises while chroma decreases. This relationship suggests a positive correlation between brightness and body temperature, and a negative correlation between chroma and body temperature. Analysis of skin spectral reflectance further indicates that the reflectance of visible and near-infrared light increases with rising temperature. These results suggest that body color changes in *A. carolinensis* not only serve camouflage [29] and signal transmission [30] but also meet thermoregulatory needs, which is similar to the centralian blue-tongued skink (*P. vitticeps*), which regulates body color to reduce heat absorption in high-temperature environments [22].

Previous studies have indicated that during the breeding season, the color-changing behavior of lizards primarily serves social communication [31,32], with no significant correlation to changes in body temperature [32]. This study examines the association between body color change and temperature regulation under controlled laboratory conditions. The use of precise temperature manipulation allowed us to minimize confounding variables present in natural environments. Our findings demonstrate that skin color change is primarily driven by variations in ambient temperature, aligning with the results reported by Price et al. [21]. During the non-breeding season, color change appears to be largely temperature-dependent, facilitating efficient thermoregulation through chromatic adjustment. In contrast, body color remains consistently green throughout the breeding season, suggesting a shift in functional priority toward social signaling [21]. This multifunctional integration of color change represents an adaptive strategy that balances energy conservation with reproductive demands, enabling *A. carolinensis* to meet both survival and reproductive objectives in seasonally variable temperate habitats. We note, however, that laboratory findings should be interpreted alongside field-based behavioral and thermal data to fully elucidate the ecological significance of color variation.

At different ambient temperatures, the body temperature of ectotherms changes accordingly [33]. Garrick proposed that lizards, as ectotherms, rely on external heat sources to regulate body temperature, and their body surface temperature strongly correlates with ambient temperature [34]. *A. carolinensis* is a species distributed at relatively high latitudes in the neotropical region. Its activity capacity is limited in winter (movement rate only 1.28–2.53 cm/h), leading to low efficiency of behavioral thermoregulation [20]. In our study, the body surface temperature was closer to ambient temperature than the core body temperature. The most suitable temperature for the *A. carolinensis* was around 30 °C. At this temperature, the body surface and core temperatures were closest to the ambient temperature. In contrast, at the lower temperature of 20 °C and the higher temperature of 40 °C, there was a difference between the body temperature and the ambient temperature.

Ectotherms may regulate appropriate temperature by increasing energy consumption to maintain body temperature in a relatively stable range [35]. For example, in a low-temperature environment, ectotherms would maintain body temperature by reducing heat conduction and increasing metabolic rate; in a high-temperature environment, they may reduce body temperature by changing skin color and finding shade to cool down [35,36]. Temperature affects the sensitivity of melanocytes to melanocyte-stimulating hormones, thereby influencing the quantity and extent of melanin aggregation [37]. The resulting darkening or lightening of the skin typically occurs on the surface of the back or across the entire body, which helps the body absorb or reflect heat [12]. In this study, the body color of the lizard was significantly darkened at low temperatures, which was conducive to heat absorption, and the body temperature was higher than the ambient temperature (1.66 ± 0.73 °C). At higher body temperatures, lighter body color individuals may reduce the risk of overheating due to rapid warming [13], below ambient temperature (2.84 ± 0.76 °C). The results suggest that changes in skin color may correlate with differences in radiative heat absorption across ambient temperature environments. However, the direct thermal consequences of these color shifts were not quantified in the present study and warrant further in-depth investigation.

Although some preliminary results have been obtained on skin color change and thermoregulation of *A. carolinensis* in this study, further studies are needed to reveal the internal mechanism at different temperatures. This includes an in-depth exploration of the specific physiological and molecular mechanisms of thermoregulation, such as changes in metabolite levels in the body at different temperatures and the way the nervous system controls thermoregulation. Further research is also needed to understand how *A. carolinensis* utilizes multiple thermoregulatory ways, including skin color change and behavioral regulation.

## 5. Conclusions

Our findings elucidate the relationship between body temperature and skin color in *A. carolinensis* under varying short-term ambient temperatures. The rapid color shifts in this species extend beyond their established roles in social signaling, camouflage, and stress responses to include a newly demonstrated function in thermoregulation. This work thereby offers a new perspective for understanding the integrated mechanisms of its color change.

## Figures and Tables

**Table 1:** Mean chroma, hue, and brightness of *A. carolinensis* under different environmental temperatures.

Background Color		Ambient Temperatures		
		20 °C	30 °C	40 °C
White background	Chroma (%)	43.55 ± 0.60	41.82 ± 0.60	36.43 ± 0.60
	Hue (°)	95.80 ± 0.67	93.13 ± 0.67	8.82 ± 0.68
	Brightness (cd/m^2^)	71.47 ± 0.57	82.63 ± 0.62	88.05 ± 0.67
Brown background	Chroma (%)	16.80 ± 0.30	15.98 ± 0.30	14.80 ± 0.20
	Hue (°)	33.96 ± 0.43	32.60 ± 0.28	33.40 ± 0.38
	Brightness (cd/m^2^)	70.87 ± 1.70	68.15 ± 1.96	75.74 ± 1.77

**Table 2:** Peak wavelengths and maximum skin spectral reflectance (%) of *A. carolinensis* at different ambient temperatures in a white background.

Measurement Range	Body Part		Ambient Temperatures					
			15 °C	20 °C	25 °C	30 °C	35 °C	40 °C
300–700 nm	Dorsal	Wavelength (nm)	567.17	568.93	571.85	567.76	574.19	578.87
		Reflectance (%)	26.61 ± 0.66	26.02 ± 0.58	25.66 ± 0.70	31.79 ± 0.60	30.01 ± 0.88	31.40 ± 0.66
	Head	Wavelength (nm)	569.51	571.85	574.19	571.85	580.04	584.71
		Reflectance (%)	23.62 ± 0.92	23.97 ± 0.82	21.35 ± 1.09	25.65 ± 1.22	23.46 ± 1.50	25.52 ± 1.41
700–2500 nm	Dorsal	Wavelength (nm)	1068.19	1074.79	1120.91	1107.75	1140.65	1127.5
			1284.87	1284.87	1265.26	1271.8	1265.26	1271.8
			1667.07	1660.65	1660.65	1667.07	1660.65	1660.65
			2237.24	2237.24	2237.24	2230.99	2237.24	2243.49
		Reflectance (%)	9.17 ± 0.39	9.50 ± 0.26	9.58 ± 0.24	9.91 ± 0.17	9.62 ± 0.18	10.52 ± 0.21
			10.11 ± 0.20	10.31 ± 0.18	9.42 ± 0.18	10.28 ± 0.17	9.75 ± 0.19	10.36 ± 0.18
			6.86 ± 0.15	6.65 ± 0.14	5.92 ± 0.13	6.92 ± 0.15	6.46 ± 0.16	6.69 ± 0.14
			3.74 ± 0.10	3.60 ± 0.10	3.18 ± 0.08	3.77 ± 0.09	3.46 ± 0.09	3.38 ± 0.08
	Head	Wavelength (nm)	1068.19	1074.79	1120.91	1107.75	1140.65	1127.5
			1284.87	1284.87	1265.26	1271.8	1265.26	1271.8
			1667.07	1660.65	1660.65	1667.07	1660.65	1660.65
			2237.24	2237.24	2237.24	2230.99	2237.24	2243.49
		Reflectance (%)	10.30 ± 0.67	10.17 ± 0.39	9.57 ± 0.75	10.79 ± 0.46	10.26 ± 0.45	11.64 ± 0.39
			10.33 ± 0.43	11.61 ± 0.34	8.88 ± 0.41	10.27 ± 0.34	10.20 ± 0.46	11.09 ± 0.33
			7.73 ± 0.40	7.35 ± 0.37	5.81 ± 0.33	7.36 ± 0.33	7.19 ± 0.44	7.48 ± 0.34
			5.65 ± 0.43	5.21 ± 0.44	3.92 ± 0.27	5.11 ± 0.37	5.00 ± 0.42	5.28 ± 0.37

## Data Availability

The data that support the findings of this study are available from the corresponding author upon reasonable request.

## Supplementary Materials

The following supporting information can be downloaded at: https://www.mdpi.com/article/10.3390/ani16020203/s1, Figure S1: The duration of body temperature stabilization in *Anolis carolinensis* under different ambient temperatures was recorded using an infrared thermographic camera; Figure S2: Regression plot for body temperatures of *Anolis carolinensis* measured by IRC and IRT; Figure S3: The skin color characteristics of *Anolis carolinensis* under different ambient temperatures; Figure S4: Difference between body temperature and ambient temperature at six ambient temperatures in white background; Figure S5. Comparison of body temperatures between male and female individuals on a white background under different environmental temperatures.

## Abbreviations

The following abbreviations are used in this manuscript:

MSH	Melanocyte-stimulating hormone
Vis-NIR	Visible-near-infrared
NIR	Near-Infrared
SVL	Snout–vent length
UV	Ultraviolet
CTMax	Critical thermal maximum
CTMin	Critical thermal minimum
T_cb_	Core body temperature
T_bs_	Body surface temperature
