# Supplementary material for: Skin Coloration Changes and Thermoregulation in Anolis carolinensis Across Different Thermal Environments
Source: Animals (Basel). 2026 Jan 9;16(2):203. doi: 10.3390/ani16020203 (PMC12838270; doi:10.3390/ani16020203)
Supplement: Supplementary file 1 [file animals-16-00203-s001.zip › animals-4023101-supplementary.pdf]

Skin Coloration Changes and Thermoregulation in *Anolis carolinensis* Across Different Thermal Environments

1. Thermal equilibrium time of individuals and data correlation of two infrared thermal devices

Under different ambient temperatures, the rate of skin temperature changes in individuals after exposure was observed. Within 30 minutes of exposure, the skin temperature change rate of all individuals was ⩽ 0.2°C / min (this standard is defined as achieving thermal equilibrium).

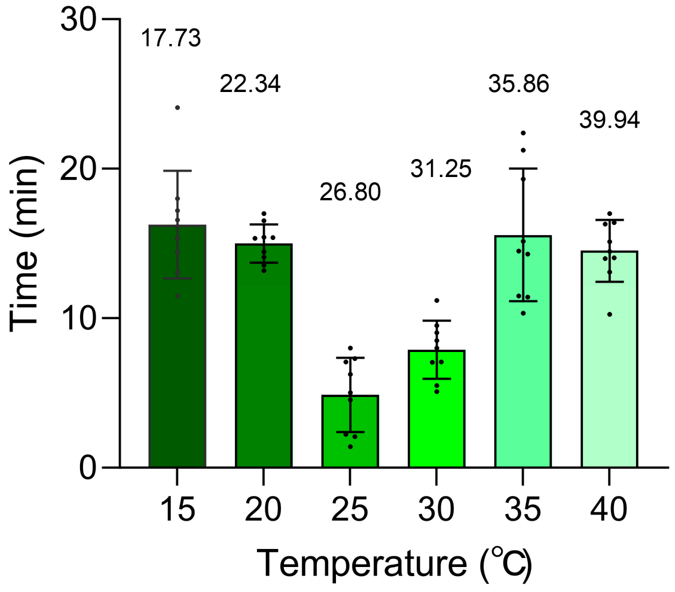

Figure S1. The duration of body temperature stabilization in *Anolis carolinensis* under different ambient temperatures was recorded using an infrared thermographic camera. In the bar chart, the dots and short bars represent the mean values and standard errors, respectively, while the numbers above indicate the body surface temperature at the time of temperature stabilization.

By comparing the data of the two devices, we observed that the average temperature of the infrared thermometer was highly correlated with the average temperature of the infrared camera (Simple linear regression, $r^2 \geq 0.95$, $p < 0.001$).

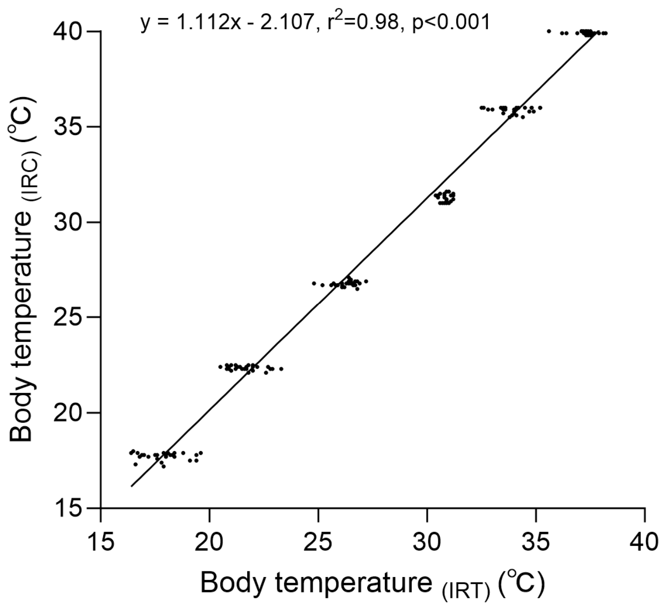

Figure S2. Regression plot for body temperatures of *Anolis carolinensis* measured by IRC and IRT. The scattered dots in the graph represent the body surface temperature measured by both instruments, and the straight line is a linear regression line.

2. **Body temperature and skin coloration at different ambient temperatures**

Under a white background, an analysis of the color space of the skin surface of the *A. carolinensis* at six temperatures (15°C, 20°C, 25°C, 30°C, 35°C, 40°C) revealed a significant correlation between changes in lightness, chromaticity, and hue values of the skin color and environmental temperature (Figure S3).

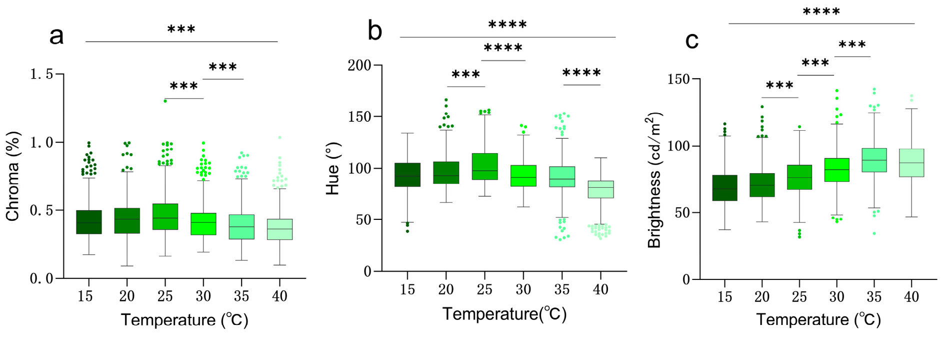

Figure S3. The skin color characteristics of *Anolis carolinensis* under different ambient temperatures. (A) Chroma values; (B) Hue values; (C) Brightness values.

At 15°C and 20°C, core body temperature and body surface temperature were significantly different from ambient temperature. With the increase of ambient

temperature, core body temperature and body surface temperature were closest to ambient temperature at 30℃. At 35℃ and 40℃, the differences between core temperature and body surface temperature increased again (Figure S4).

The body temperature of this species is closest to the ambient temperature at 30℃, indicating that 30℃ has significant biological importance for this species. Further analysis of the difference between body surface temperature and ambient temperature revealed that there were no significant differences between the temperature differences at 25℃ and 30℃, and 30℃ and 35℃ ($p=0.478$, $p=1.000$); however, significant differences were observed between 20℃ and 30℃, and 30℃ and 40℃ ($p=0.012$, $p<0.001$) (Figure S4). In addition, we compared the coloration indices of this species at 15℃ and 20℃, and the results showed no significant differences in chroma, hue, and brightness (Figure S3). Based on the analysis of body temperature differences and coloration indices, this study ultimately selected three environmental temperatures—20℃ (cold extreme), 30℃ (preferred temperature), and 40℃ (hot extreme)—for subsequent experiments.

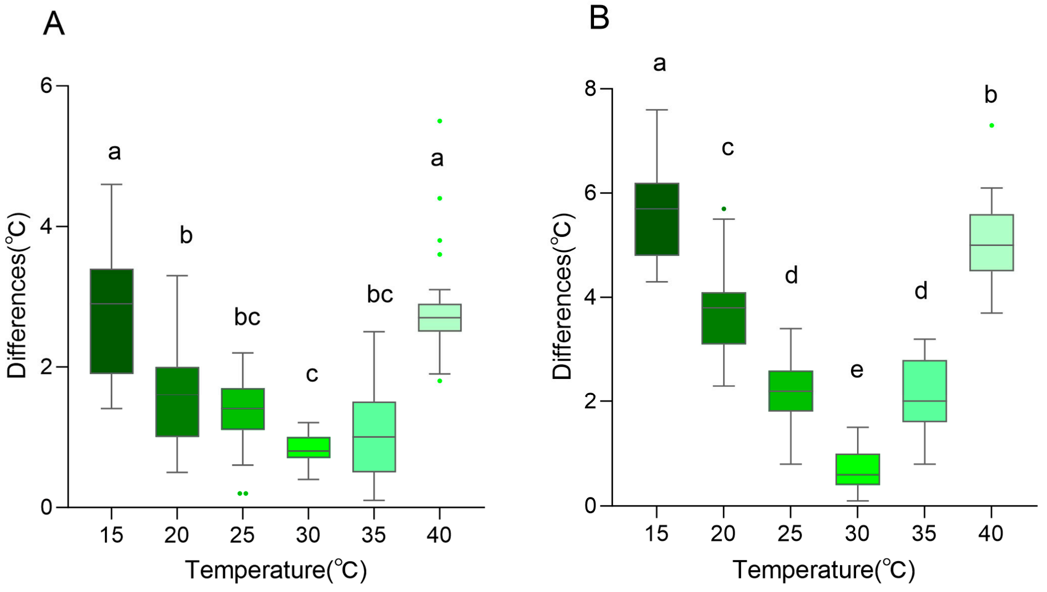

Figure S4. Difference between body temperature and ambient temperature at six ambient temperatures in white background. (A) Differences between body surface temperature and ambient temperature; (B) Differences between core body temperature and ambient temperature.

Under different environmental temperature gradients, there were no statistically significant differences in both the body surface temperature and core temperature

between female and male individuals (p>0.05), indicating that the two sexes have a consistent pattern of body temperature response to changes in environmental temperature (Figure S5).

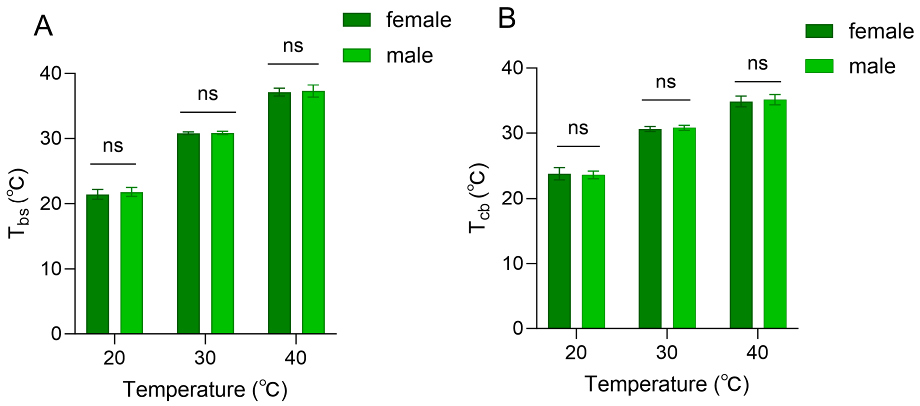

Figure S5. Comparison of body temperatures between male and female individuals on a white background under different environmental temperatures. (A) Comparison of body surface temperature between male and female individuals; (B) comparison of core temperature between male and female individuals.
